# Serum biomarker analysis may guide management of anemia in patients with chronic liver disease

**DOI:** 10.3389/fmed.2026.1797978

**Published:** 2026-04-29

**Authors:** Yihui Rong, Wenpeng Fu, Yanyu Jiang, Xudong Gao, Jie Han

**Affiliations:** 1 Department of Infectious Diseases, Peking University International Hospital, Beijing, China; 2 Department of Infectious Diseases, Zhaoyuan People's Hospital, Yantai, Shandong, China; 3 Department of Hepatology, The 5th Medical Center of PLA General Hospital, Beijing, China; 4 Department of Hepatology, Qilu Hospital, Cheeloo College of Medicine, Shandong University, Jinan, Shandong, China

**Keywords:** anemia, biomarkers, chronic liver disease, correlation analysis, hepatic function

## Abstract

**Background:**

Chronic liver disease (CLD) is a significant global health threat and has emerged as one of the leading causes of mortality worldwide. Anemia is a prevalent complication observed in patients with CLD, with 75% of these patients being susceptible to the condition. The utility of anemia-associated biomarkers for the diagnosis and management of this condition remains inadequately defined. In this study, we collected hematological data from patients with CLD and analyzed a panel of anemia-related biomarkers, aiming to investigate the correlations between these markers in patients with anemia secondary to chronic liver disease.

**Methods:**

The first cohort of 119 patients from the Department of Hepatology at Qilu Hospital of Shandong University was recruited for the study. Demographic data of the patients were included and ANOVA analysis based on anemia types was conducted. A subset of 64 patients with available serum samples for hepcidin measurement was included as a second cohort for downstream analysis. The model for end-stage liver disease (MELD), aspartate aminotransferase to platelet ratio index (APRI), and fibrosis-4 (FIB-4) scores were used to evaluate liver functions. The correlation of these markers was also calculated. SPSS software and R program were utilized to perform statistical analysis and plot graphs.

**Results:**

This study indicates that anemia in CLD is closely associated with disease severity and related complications. We found that in patients with macrocytic and normocytic anemia, the level of erythropoietin (EPO) was positively correlated with soluble transferrin receptor (STFR). While mean corpuscular volume (MCV) was positively correlated with MELD and APRI scores, total bilirubin (TBIL) was positively correlated with FIB-4 scores. WBC and ferritin were positively correlated. Additionally, various biomarkers were statistically significant across macrocytic, normocytic, and microcytic anemia groups, as well as different groups by the bilirubin level.

**Conclusion:**

Anemia in chronic liver disease should not be overlooked. This exploratory study suggests that anemia-related biomarkers may hold promise for evaluating liver function and could inform the management of anemia in these patients, though future validation in larger cohorts is necessary to confirm these preliminary findings and establish their clinical utility.

## Introduction

Chronic liver disease (CLD) affects approximately 1.5 billion individuals globally (1), which encompasses a spectrum of hepatic pathologies, including chronic hepatitis, cirrhosis, and liver cancer. Different etiological factors are found to be the drivers of liver disease, such as hepatitis B infection, hepatitis C infection, lifestyle, ethanol consumption, etc. (2, 3). Significantly, due to the fundamental roles of the liver in iron metabolism, red blood cell life-cycle, and the interactions between the liver and other body parts, 75% of chronic liver disease patients are susceptible to anemia (4, 5). The majority of such patients, however, could not be managed properly regarding their anemic status, largely due to the lack of precise diagnosis of their anemic type and deep understanding of the mechanisms causing anemia.

Some types of anemia show clear biomarkers and appear to be easier to diagnose. Macrocytic anemia can be caused by malnutrition and malabsorption of VitB12 and folic acid (6). Iron deficiency anemia can be detected in patients with microcytic anemia and confirmed iron insufficiency as determined by serum transferrin (TF) or ferritin depletion (7). Renal anemia can be diagnosed in patients with normocytic anemia and chronic renal disease stage 3b or above (8). Hemolytic anemia can be diagnosed in patients with normocytic anemia who have either decreased haptoglobin or a large increase in unconjugated bilirubin (9). In patients with hypochromic and microcytic anemia and dramatically high ferritin levels, anemia was linked to chronic inflammation (10).

While anemic patients, as described above, are commonly encountered in clinical settings, the diagnostic criteria and indicators for anemia remain limited and require further refinement. Particularly, the etiology of anemia in liver disease is particularly diverse, and most patients are diagnosed based on multiple factors or a combination of these factors. The leading cause of anemia is iron deficiency, and treatments include oral and intravenous iron therapy (11). However, other causes are also reported to exacerbate anemia including blood loss from gastric antral vascular ectasia, portal hypertensive gastropathy, peptic ulcers, etc. (12). Furthermore, the clinical application of iron therapy is often ineffective, which further illustrates the involvement of other factors, such as cirrhosis, variceal bleeding, coagulation disorders in liver diseases, and so on. Thus, it remains challenging to diagnose the type of anemia in chronic liver disease patients and the management guideline is largely missing for most patients.

Although previous studies have established general associations between anemia, iron dysregulation, inflammation, and CLD severity (4–6), a systematic analysis stratifying these complex interactions by specific anemia subtypes is lacking. To address this gap, the present study aimed to systematically investigate the relationships between anemia-associated biomarkers, erythropoietic regulators, and liver disease severity in patients with chronic liver disease. In particular, we focused on evaluating the association between erythropoietin (EPO), iron metabolism markers, inflammatory indicators, and red blood cell morphology in order to better understand the heterogeneous mechanisms underlying anemia in CLD and explore potential biomarkers that may assist in clinical stratification and management. We aimed to evaluate the frequency and causative factors of anemia, classify cases based on red blood cell (RBC) morphology in relation to hepatic abnormalities, and identify deviations from classical laboratory outcomes. By systematically correlating iron, inflammatory, and erythropoietic biomarkers across different anemia phenotypes, this work explores their distinct pathophysiological mechanisms and clinical correlations.

## Materials and methods

### Patient population

**Figure 1:**
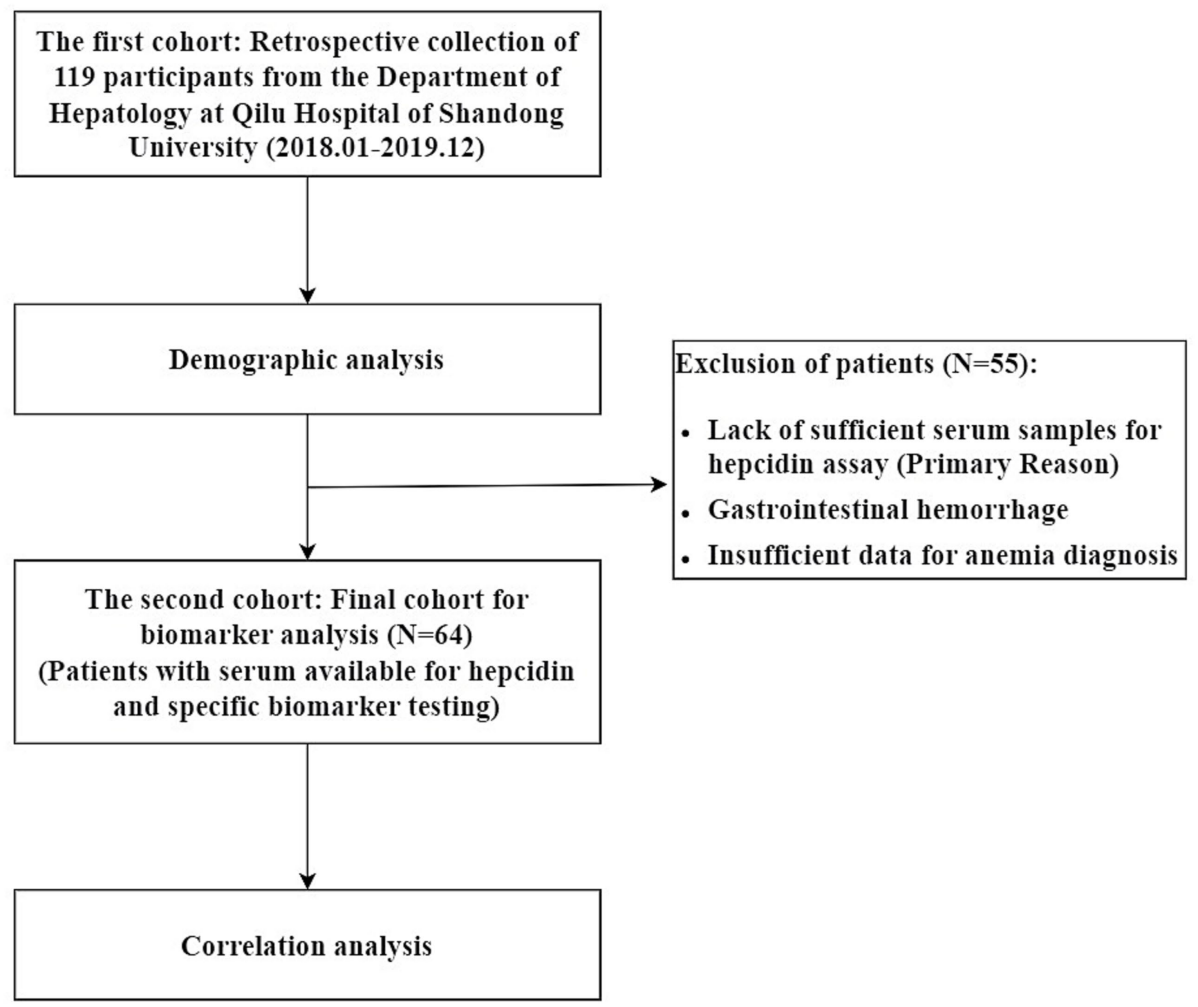
Flow chart of the study.

In this study, we examined a cohort of 119 patients from the Department of Hepatology at Qilu Hospital of Shandong University who were 15 years or older diagnosed with anemia (Male, hemoglobin less than 130 g/L; Female, hemoglobin less than 120 g/L) ^[13]^secondary to chronic liver disease between January 2018 and December 2019 in Figure 1. Diagnosis of chronic liver disease was established based on clinical assessments and laboratory findings indicative of hepatocellular damage. Patients presented with symptoms including jaundice, ascites, hypoalbuminemia, encephalopathy, elevated serum transaminase levels, prolonged prothrombin time, portal hypertension, and cirrhosis, as confirmed by imaging studies (ultrasonography, computed tomography, or magnetic resonance imaging). The etiological classification of chronic liver disease is detailed in Table 1. Patients with concurrent malignant tumors in organs other than the liver or those receiving medications that could induce or exacerbate anemia were excluded from the study. The inclusion and exclusion criteria are detailed in Supplementary Figure 1. This research was approved by the Peking University International Hospital ethics committees (2019-069), and was carried out in accordance with the Declaration of Helsinki. Given the retrospective nature of the study, the informed consents were waived.

**Table 1:** Demographics of the patients.

Characteristic	Number of patients (*N* = 119)	Percentage (%)
Age		
<30	2	1.68%
30–60	72	60.50%
>60	45	37.82%
Gender		
Male	83	69.75%
Female	36	30.25%
Underlying diseases		
Alcohol	34	28.58%
Hepatitis B infection	63	52.94%
Hepatitis C infection	2	1.68%
Autoimmune hepatitis	7	5.88%
Primary biliary cholangitis	6	5.04%
Others*	7	5.88%
Splenomegaly		
Splenomegaly	108	90.8%
(with hypersplenia)	88	(73.95%)
No splenomegaly	11	9.24%
Liver cancer		
Liver cancer	32	26.89%
No liver cancer	87	73.11%
Signs of portal hypertension		
Hypertension	111	93.28%
(Ascites)	76	(63.87%)
(Esophagogastric varices)	35	(29.41%)
No hypertension	8	6.72%
Size of the cell		
Large	35	29.41%
Normal	71	59.66%
Small	13	10.92%
Kidney function		
BUN-High	19	15.97%
BUN-Normal	78	65.55%
BUN-Low	22	18.49%
CR-High	8	6.72%
CR-Normal	98	82.35%
CR-Low	13	10.92%
Hepatic encephalopathy (total counted: 64)		
Hepatic encephalopathy	3	4.69%
Elevated ammonia	4	6.25%
Bilirubin		
Elevated	91	76.47%
Normal	28	23.53%
VitB12		
High	75	63.02%
Normal	42	35.29%
Low	2	1.68%
Folic acid		
High	7	5.88%
Normal	97	81.51%
Low	15	12.61%

^*^Others include: drug-induced, pancreatic cancer, idiopathic, hepatolenticular degeneration.

### Data collection and laboratory analysis

Patients with gastrointestinal hemorrhage and those lacking sufficient data for an anemia diagnosis were excluded from the analysis. From the initial cohort of 119 patients, 64 patients who had available serum samples necessary for measuring hepcidin and other specific biomarkers (e.g., STFR, IL-6) were included for the detailed biomarker analysis. The primary reason for excluding 55 patients was the lack of sufficient serum for these specific assays. No imputation was performed for missing biomarker data; analyses were conducted based on available data. The flow of patient selection is detailed in Figure 1. The availability of serum specimens was the determining factor for inclusion in the comprehensive biomarker correlation analysis. Patients without sufficient serum for hepcidin measurement were excluded from this specific part of the study. We collected data on various inflammatory factors, hepcidin, ferritin, and several anemia-related indicators, and conducted a correlation analysis. These include blood count, serum iron, serum vitamin B12 (VitB12), folate levels, serum ferritin, serum hepcidin, bilirubin, haptoglobin (HP), total iron binding capacity (TIBC), erythropoietin (EPO), aspartate aminotransferase (AST), alanine aminotransferase (ALT), soluble transferrin receptor (STFR), interleukin-6 (IL-6), interleukin 1 (IL-1), TNF-*α*, prothrombin time (PT), total protein (TP), TF, gamma-globulin (GLB), albumin (ALB), fibrinogen, blood urea nitrogen (BUN), creatinine (CR), procalcitonin test (PCT), mean corpuscular volume (MCV), international normalized ratio (INR). Serum hepcidin levels were measured using a commercially available ELISA Kit (CUSABIO, China). All other biochemical parameters were analyzed using automated clinical chemistry analyzers (R&D systems, USA) in the central laboratory of Qilu Hospital of Shandong University.

### Classification of anemia and liver disease score

Anemia can be categorized into mild (Male, HB 110-129 g/L; Female, HB 110-119 g/L), moderate (HB 80-109 g/L), and severe (HB lower than 80 g/L) based on hemoglobin levels (13). Additionally, it can be classified as macrocytic (MCV > 100 fL), normocytic (MCV 80–100 fL), or microcytic (MCV < 80 fL) anemia according to MCV values (14). In accordance with the World Health Organization’s standard for diagnosing iron deficiency anemia (IDA) (15), we classify patients with anemia secondary to chronic liver disease as having IDA if their serum ferritin levels are below 70 μg/L. Patients with serum vitamin B12 levels below 157 pg./mL were identified as having vitamin B12 deficiency (16). Similarly, patients with folate levels below 5.3 ng/mL were identified as having folate deficiency (17). A diagnosis of splenic hyperfunction is established for patients presenting with splenomegaly accompanied by pancytopenia (18). Moreover, pancytopenia was diagnosed in patients having low white blood cell count (less than 4 × 10^9/L) and thrombocytopenia with less than 100 × 10^9/L (19). The liver disease assessments used in this study include the Model for MELD score and liver fibrosis diagnostic scores, specifically the APRI score and the FIB-4 score (20). The calculation of each liver disease score is as follows:

MELD score calculation (21): R = 3.8 × ln (TBIL) + 11.2 × ln(INR) + 9.6 × ln (CR) + 6.43 × X.

(X = 0: Liver pathogeny are PBC or alcohol, X = 1: Liver pathogeny are others).

APRI score calculation (22): APRI = (AST÷ (upper limit of normal) × 100)/PLT.

FIB4 score calculation (23): FIB-4 = (Age × AST) ÷ (PLT × $\sqrt{\mathrm{ALT}}$ ).

### Statistics

Statistical analyses of the obtained results were conducted using R program (V.4.3.0)^1^ and SPSS software (V.25.0, IBM). Specifically, the R packages “ggplot2” and “ggstatsplot” were employed for data visualization and statistical evaluation. The variables were compared using Analysis of Variance (ANOVA) and the Kruskal-Wallis test, with ANOVA applied under the assumption of homogeneity of variances. In cases of unequal variances, the Welch test was utilized. Additionally, Spearman’s correlation analysis was performed to assess the relationships between the factors. Results with two-sided *p*-value less than 0.05 were deemed statistically significant.

## Results

### Demographics of patients

Demographic data for the 119 patients diagnosed with anemia are presented in Table 1. Among these patients, 1.68% were under 30 years of age, 60.5% were between 30 and 60 years old, and 37.82% were over 60 years old. The cohort comprised 69.75% males and 30.25% females. With respect to underlying conditions, 28.58% of patients were diagnosed with alcohol-related liver disease, 52.94% were infected with hepatitis B virus, and 26.89% were affected by liver cancer. Furthermore, 1.68% contracted hepatitis C infection, 5.88% had autoimmune hepatitis, 5.04% exhibited primary biliary cholangitis, and 5.88% presented with other underlying conditions. Notably, 90.8% of patients exhibited splenomegaly, with 73.95% of these individuals also presenting with hypersplenism. Furthermore, 93.28% of patients demonstrated signs of portal hypertension, of which 63.87% presented ascites and 29.41% had esophagogastric varices. Hepatic encephalopathy was observed in 4.69% of patients, while 6.25% exhibited elevated ammonia levels. Elevated bilirubin values were found in 76.47% of patients, whereas 23.53% maintained normal bilirubin levels.

We analyzed hematological parameters and nutritional factors associated with anemia in our patient cohort. The results revealed that 29.41% of patients exhibited macrocytic anemia, while 59.66% presented with normocytic anemia, and 10.92% had microcytic anemia. In terms of renal function, 15.97% of patients exhibited elevated BUN levels, while 6.72% showed increased CR levels. Concerning nutritional status, only 1.68% of patients had low VitB12 levels, and 12.61% exhibited low folic acid levels. These findings indicate that anemia in patients with liver disease is primarily due to the underlying hepatic condition, with only minor contributions from renal dysfunction or nutritional deficiencies.

For the first cohort, the mean and standard deviation for each variable are presented in Supplementary Table 1. Similarly, Supplementary Table 2 provides the descriptive statistics, including the mean and standard deviation, for the subsequent cohort.

### Correlation of various Fe indicators and EPO

**Figure 2:**
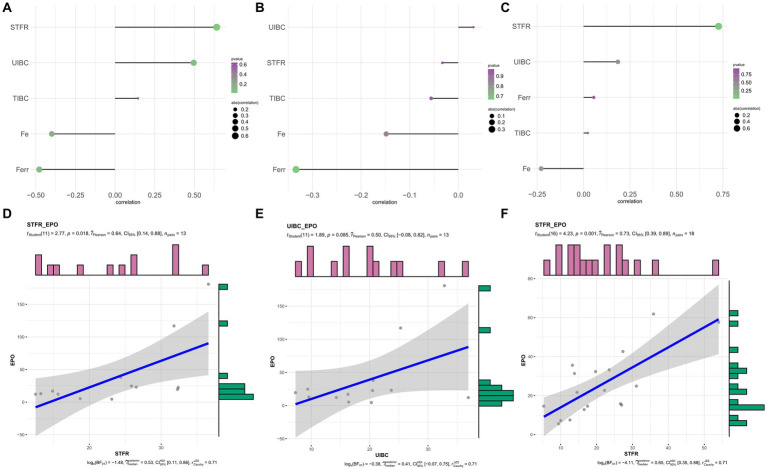
Correlations between iron-related biomarkers and erythropoietin (EPO) across anemia subtypes. **(A–C)** Correlation spot showing the correlation coefficient (r) and statistical significance between EPO and iron indicators (Fe, serum iron; UIBC, unsaturated iron-binding capacity; TIBC, total iron-binding capacity; STFR, soluble transferrin receptor; Ferr, ferritin) in patients with **(A)** macrocytic, **(B)** microcytic, and **(C)** normocytic anemia. The size of the circles and the color intensity represent the strength and direction of the correlation, as indicated by the scale. **(D–F)** Scatter plots with linear regression lines (blue) and 95% confidence intervals (shaded area) illustrating the significant correlations between STFR and EPO in **(D)** macrocytic, **(F)** normocytic anemia groups and **(E)** UIBC and EPO in macrocytic anemia group.

To exclude patients with complications related to bleeding or other issues, we selected a cohort of 64 patients for downstream biomarker analysis. According to the value of MCV, patients were categorized into three groups: microcytic anemia (MCV < 80 fL), normocytic anemia (MCV 80–100 fL), and macrocytic anemia (MCV > 100 fL). To investigate the potential influence of various biomarkers on EPO production, we analyzed the relationships between several iron indicators (serum iron, unsaturated iron-binding capacity (UIBC), TIBC, ferritin, and STFR) and EPO. In the macrocytic anemia group, STFR, UIBC, and TIBC showed positive correlations with EPO, whereas serum iron and ferritin showed negative correlations (Figure 2A). However, only the correlation with STFR was statistically significant (*r* = 0.641, *p* = 0.018). (Figure 2D). In the microcytic anemia group, no statistically significant correlations were observed between EPO and the measured iron indicators (all *p* > 0.05), though UIBC showed a non-significant positive association (Figures 2B,E). In the normocytic anemia group, iron indicators that displayed a trend of positive correlation with EPO included STFR, UIBC, ferritin, and TIBC, whereas serum iron showed a trend of negative correlation with EPO (Figure 2C). Notably, the correlation coefficient for STFR with EPO was statistically significant, with *r* = 0.727 and *p* = 0.0006 (Figure 2F) (Table 2). In the macrocytic and normocytic anemia groups, STFR and EPO demonstrated a statistically significant positive correlation; however, this association was absent in the microcytic anemia group.

**Table 2:** Correlation of various Fe indicators and EPO.

Anemia types	Symbol	Correlation	*p* value
Macrocytic anemia	Fe	−0.399	0.176
	UIBC	0.496	0.085
	TIBC	0.144	0.638
	STFR	0.641	0.018
	Ferr	−0.478	0.099
Normocytic anemia	Fe	−0.228	0.362
	UIBC	0.184	0.464
	TIBC	0.022	0.930
	STFR	0.727	0.0006
	Ferr	0.055	0.829
Microcytic	Fe	−0.148	0.852
	UIBC	0.031	0.969
	TIBC	−0.056	0.944
	STFR	−0.033	0.967
	Ferr	−0.334	0.666

### Relationship between ferritin, hepcidin, and anemia types

**Figure 3:**
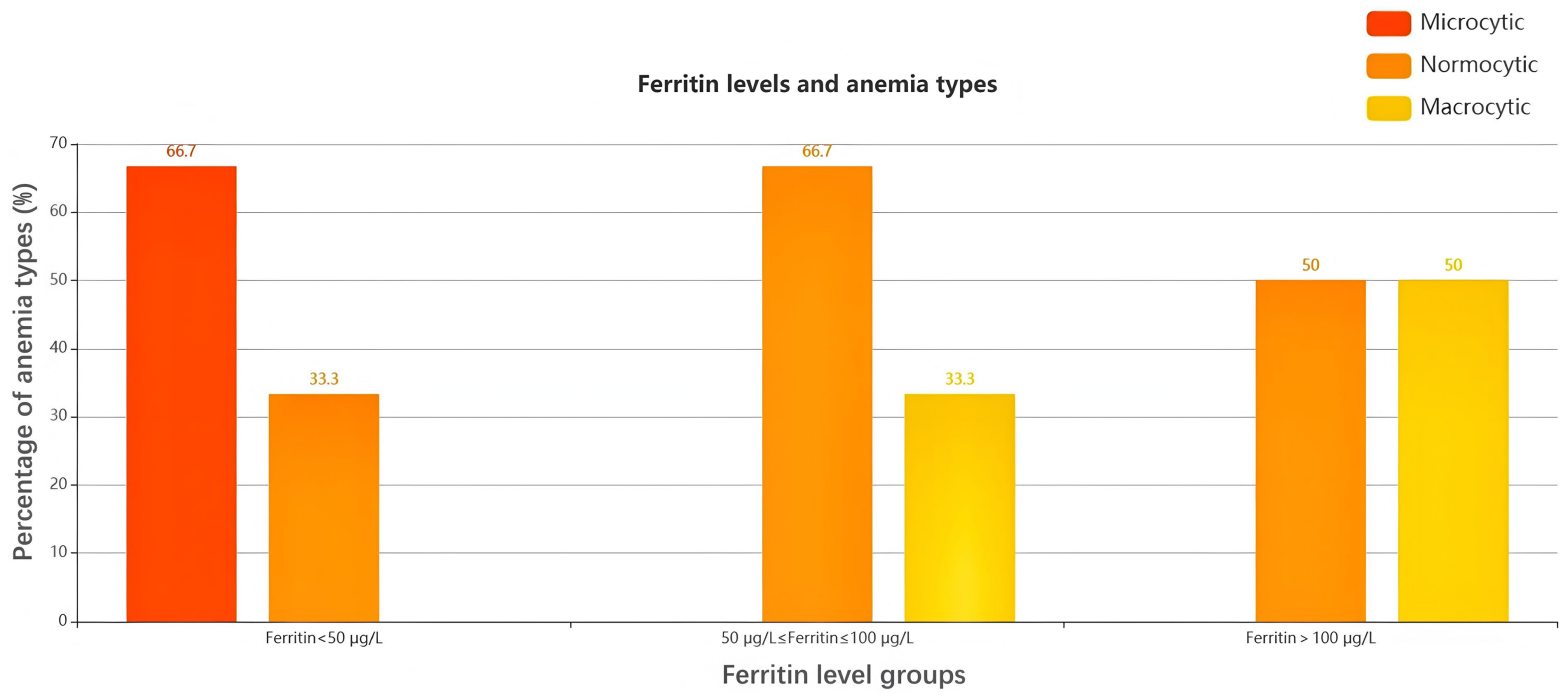
Ferritin levels and anemia types.

Ferritin serves as a key indicator of iron storage levels in the body, prompting an investigation into whether iron-related biomarkers differ when categorizing anemic patients based on ferritin levels. In patients with ferritin levels below 50 μg/L, microcytic anemia was observed in 66.7% of cases, while normocytic anemia accounted for 33.3%. For patients with ferritin levels ranging from 50 to 100 μg/L, normocytic anemia comprised 66.7%, and macrocytic anemia represented 33.3%. In the group with ferritin levels exceeding 100 μg/L, both normocytic and macrocytic anemia were equally represented, each constituting 50%. This biased distribution pattern suggests that ferritin levels may correlate with RBC morphology in patients with CLD, aligning with findings that associate ferritin levels with RBC morphology in other conditions, such as Alzheimer’s disease (24). However, due to the limited sample size, the correlation of hepcidin levels across different ferritin and anemia groups did not yield statistically significant results, with *p*-values exceeding 0.05 for both ferritin groups and anemia types (Figure 3; and Supplementary Figure 2).

### Correlation between inflammation factors and hepcidin, ferritin, and anemia types

**Figure 4:**
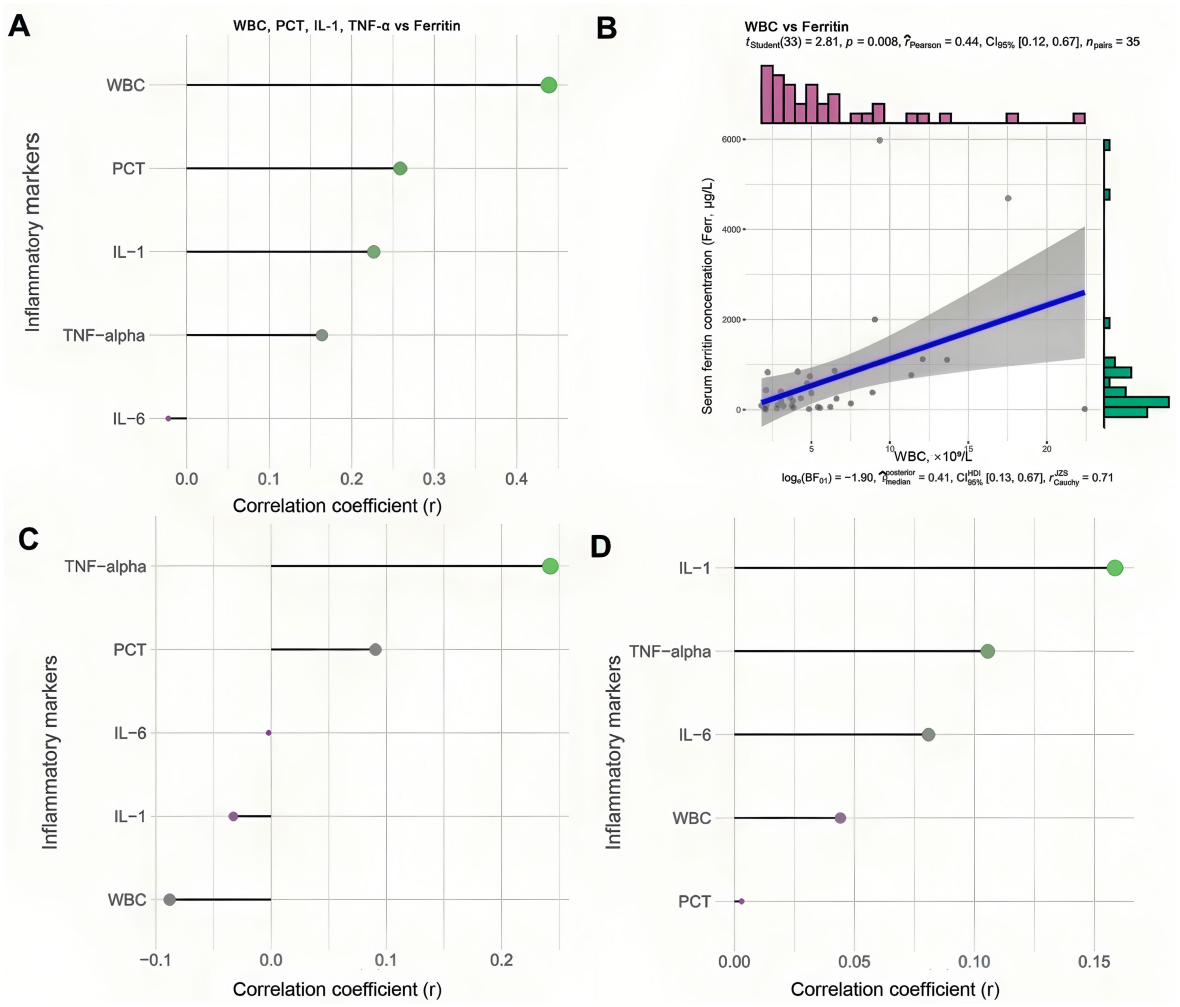
Associations between inflammatory markers, iron regulatory hormones, and anemia subtypes. **(A)** Correlation spot illustrating the relationships between various inflammatory markers (WBC, white blood cell count; PCT, procalcitonin; IL-1, interleukin-1; TNF-*α*, tumor necrosis factor-alpha) and serum ferritin levels. **(B)** Scatter plot of the significant correlation between WBC and ferritin, with the regression line and 95% confidence interval. **(C)** Correlation spot showing the relationships between the same inflammatory markers and serum hepcidin levels. **(D)** Correlation spot displaying the correlations between inflammatory markers and across anemia types.

Given the critical role of inflammation in anemia, particularly in cases characterized by functional iron deficiency, we assessed the correlation between various inflammatory markers and hepcidin, ferritin, and different types of anemia. Our analysis revealed a positive correlation between WBC count, PCT, IL-1, and TNF-*α* with ferritin levels, as illustrated in Figure 4A. Notably, the correlation coefficient for WBC with ferritin was 0.439, with a *p*-value of less than 0.05, as shown in Figure 4B. Figure 4C indicates that TNF-α and PCT exhibited a positive relationship with hepcidin; however, these differences did not reach statistical significance (*p* > 0.05). Furthermore, Figure 4D demonstrates that IL-1, TNF-α, IL-6, and WBC were positively correlated with various types of anemia, although the *p*-values exceeded 0.05 (Supplementary Table 2). Ferritin levels exhibit a significant positive correlation with inflammatory factors. Similarly, hepcidin and different types of anemia are also positively correlated with these inflammatory markers.

### Association between hematological indicators and liver function scores

**Figure 5:**
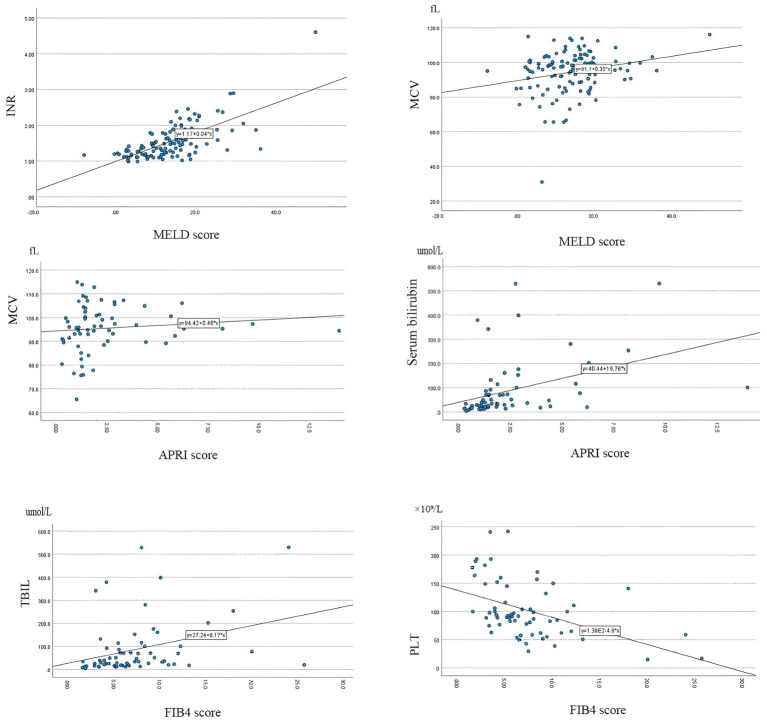
Associations between hematological parameters and liver disease severity scores. Scatter plots depict relationships between mean corpuscular volume (MCV), total bilirubin (TBIL), procalcitonin (PCT), international normalized ratio (INR), platelet count (PLT) and the Model for End-Stage Liver Disease (MELD) score, Aspartate Aminotransferase to Platelet Ratio Index (APRI), and Fibrosis-4 (FIB-4) index. Solid lines represent the Spearman regression fit.

To assess the impact of liver function and various hematological parameters, including anemia biomarkers, we calculated the MELD, APRI, and FIB-4 scores. Spearman’s correlation analysis revealed a positive correlation between the MELD score and MCV. Similarly, the APRI score demonstrated a positive correlation with TBIL and MCV. Furthermore, the FIB-4 score exhibited a positive correlation with TBIL (Figure 5).

### Analysis of variance

In the analysis of the first cohort, participants were categorized based on the type of anemia: macrocytic, normocytic, and microcytic. The results of the analysis of variance are presented in Table 3. Levene’s test was conducted to assess the equality of variances. Among the indicators with equal variances, hemoglobin (HB), RBC, MCV, mean corpuscular hemoglobin (MCH), mean cell hemoglobin concentration (MCHC), red blood cell distribution width (RDW-CV), and TIBC demonstrated statistically significant differences (*p* < 0.05). Conversely, among the indicators with unequal variances, plasma thromboplastin antecedent (PTA), BUN, PLT, UIBC, STFR, and bilirubin levels were also found to be statistically significant (*p* < 0.05).

**Table 3:** ANOVA analysis of the first cohort.

Levene’s test for equality of variances: variances equal					
Dependent variable	Type III sum of squares	df	Mean square	*F*	Sig.
HB	4025.731	2	2012.865	7.740	<0.001
RBC	8.226	2	4.113	14.402	<0.001
MCV	9137.609	2	4568.805	169.757	<0.001
MCH	1641.188	2	820.594	139.005	<0.001
MCHC	14737.233	2	7368.616	33.437	<0.001
RDW-CV	103.749	2	51.874	4.515	0.013
TIBC	5183.841	2	2591.920	18.177	<0.001

Variances unequal				
Dependent variable	Statistic	df1	df2	Sig.
PTA	4.496	2	39.109	0.017
BUN	8.097	2	64.341	<0.001
PLT	3.318	2	31.270	0.049
UIBC	31.332	2	34.035	<0.001
STFR	9.445	2	29.379	<0.001
Bilirubin	4.522	2	51.454	0.016

### Expression of various biomarkers stratified by anemia types and bilirubin levels

In the second cohort, when categorized by macrocytic, normocytic, and microcytic anemia, significant differences were observed among the variance-equal indicators, including HB, hematocrit test (HCT), MCV, MCH, MCHC, PT, PT-INR, and PTA (*p* < 0.05, Table 4). Additionally, among the variance-unequal indicators, TBIL and DBIL levels were statistically significant (*p* < 0.05, Table 4). The normocytic anemia group exhibited the highest levels of TBIL and DBIL, whereas the microcytic anemia group demonstrated the lowest levels of these bilirubin parameters (Supplementary Table 3).

**Table 4:** Expression of various indicators when grouped by MCV.

Equal variances					
Dependent variable	Type III sum of squares	df	Mean square	*F*	Sig.
HB	6804.78	2	3402.39	7.067	0.002
HCT	359.015	2	179.508	4.488	0.015
MCV	4905.682	2	2452.841	113.666	<0.001
MCH	792.925	2	396.463	86.133	<0.001
MCHC	7055.794	2	3527.897	21.135	<0.001
PT	136.645	2	68.323	4.116	0.021
PT-INR	0.986	2	0.493	3.847	0.027
PTA	2858.812	2	1429.406	4.984	0.010

Unequal variances				
Dependent variable	Statistic	df1	df2	Sig.
TBIL	6.877	2	36.966	0.003
DBIL	10.088	2	35.211	<0.001

When stratified by bilirubin levels—specifically, normal bilirubin levels, levels ranging from 1 to 2 times the normal range, and levels exceeding 2 times the normal range—statistically significant differences (*p* < 0.05) were observed among the indicators with equal variances, including MCH, MCHC, PT-INR, PTA, and FIB (Table 5). Conversely, for the indicators exhibiting unequal variances, PT, ALT, AST, AKP, TBIL, DBIL, and indirect bilirubin (IBIL) demonstrated statistically significant differences (*p* < 0.05, Table 5).

**Table 5:** Expression of various indicators when grouped by bilirubin levels.

Equal variances					
Dependent variable	Type III sum of squares	df	Mean square	*F*	Sig.
MCH	115.945	2	57.973	3.791	0.028
MCHC	1780.101	2	890.050	3.771	0.029
PT-INR	1.75	2	0.875	7.581	0.001
PTA	4825.965	2	2412.983	9.575	<0.001
FIB	9.359	2	4.679	5.824	0.005

Unequal variances				
Dependent variable	Statistic	df1	df2	Sig.
PT	7.423	2	40.203	0.002
ALT (IU/L)	4.651	2	33.799	0.016
AST (IU/L)	9.126	2	31.379	<0.001
AKP (IU/L)	3.995	2	40.119	0.026
TBIL (umol/L)	55.706	2	37.774	<0.001
DBIL (umol/L)	27.956	2	30.248	<0.001
IBIL (umol/L)	30.898	2	36.269	<0.001

In Table 6, when analyzed based on HB reduction alone, as well as in conjunction with reductions in PLT or WBC counts, and the presence of pancytopenia, the following indicators demonstrated statistical significance (*p* < 0.05) among those with equal variance: HP, UIBC, TIBC, TF, IL-6, and FIB. Conversely, among the indicators exhibiting unequal variance, DBIL and PCT were also found to be statistically significant (*p* < 0.05).

**Table 6:** Expression of various indicators when grouped by anemia types.

Equal variance					
Dependent variable	Type III sum of squares	df	Mean Square	*F*	Sig.
HP	1.141	2	0.571	3.942	0.029
UIBC	1883.174	2	941.587	3.445	0.044
TIBC	1297.943	2	648.972	4.489	0.019
TF	2.34	2	1.17	3.52	0.042
IL-6	292.088	2	146.044	7.903	0.002
FIB	2.446	2	1.223	4.347	0.021

Unequal variance				
Dependent variable	Statistic	df1	df2	Sig.
PCT	5.890	2	13.802	0.014

## Discussion

Our study provides a comprehensive analysis of the interplay between anemia-related biomarkers and liver disease severity in patients with chronic liver disease (CLD). The principal finding is that the relationships between key regulators of iron metabolism and erythropoiesis, particularly erythropoietin (EPO) and soluble transferrin receptor (STFR), are dependent on red blood cell morphology. Specifically, we identified a significant positive correlation between EPO and STFR in macrocytic and normocytic anemia, which was absent in microcytic anemia. This suggests that distinct pathophysiological mechanisms underlie different anemia subtypes in CLD. In addition, routine hematological parameters such as mean corpuscular volume (MCV), together with liver function indicators including bilirubin, were significantly associated with established liver disease severity scores (MELD, APRI, FIB-4), further supporting a close link between anemia phenotype and hepatic dysfunction.

While the multifactorial nature of anemia in CLD is well-established, our findings underscore the potential value of a biomarker-guided approach to disentangle its predominant causes in individual patients (25). The significant correlations observed in this study—such as between EPO and STFR in macrocytic and normocytic anemia, and between ferritin and WBC—suggest that distinct mechanistic pathways (e.g., impaired erythropoiesis, functional iron deficiency, or inflammation) may be inferred by specific biomarker patterns (26, 27). Consequently, reliance on a single marker is unlikely to provide sufficient diagnostic insight (28). For instance, our data indicate that in patients with elevated ferritin and WBC levels, anemia may be predominantly driven by inflammation, in which case indiscriminate iron supplementation should be avoided (29). Conversely, the combination of elevated STFR and relatively insufficient EPO levels may suggest impaired erythropoietic compensation and could potentially identify patients who might benefit from erythropoiesis-stimulating agents (30). Therefore, a stratified interpretation of multiple biomarkers, particularly when considered together with RBC morphology, may contribute to a more precise evaluation and management of anemia in patients with CLD (31). Nevertheless, the clinical use of erythropoiesis-stimulating agents in patients with chronic liver disease should be approached with caution. Previous studies have reported that inappropriate administration of erythropoietin may increase the risk of thrombotic complications, particularly in patients with advanced liver disease or hypercoagulable states (32). Accordingly, identifying patient subgroups who are most likely to benefit from erythropoietin therapy through biomarker-guided stratification may help balance potential therapeutic benefits against associated risks.

Our findings place the pathophysiology of anemia in CLD within the established framework of inflammatory-driven anemia, while also highlighting important disease-specific nuances. The observed positive correlation between WBC and ferritin is consistent with inflammation-mediated iron dysregulation, in which pro-inflammatory cytokines (e.g., TNF, IL-6, IL-1) stimulate hepcidin production, leading to iron sequestration and functional iron deficiency (10). At the molecular level, hepcidin expression is primarily regulated through the bone morphogenetic protein (BMP)–SMAD signaling pathway (33). In hepatocytes, BMP ligands activate SMAD phosphorylation, leading to transcription of the HAMP gene encoding hepcidin, a process further enhanced by co-receptors such as hemojuvelin. In parallel, inflammatory signaling pathways, particularly IL-6-mediated JAK–STAT activation, further upregulate hepcidin expression (34).

However, in the context of CLD, our data suggest a more complex regulatory balance. Despite the inflammatory milieu, impaired hepatocellular function may compromise hepcidin synthesis, while the significant positive correlation between EPO and STFR in macrocytic and normocytic anemia indicates a compensatory erythropoietic drive (28, 35). This erythropoietic activity may further suppress hepcidin via erythroferrone-mediated mechanisms, creating a dynamic interplay between inflammation, hepatic dysfunction, and erythropoiesis (10). Consequently, iron availability in CLD patients likely reflects the net effect of these competing signals rather than a single dominant pathway. In addition, the observed associations between bilirubin levels and hematological parameters suggest that bilirubin may function as a composite biomarker reflecting hemolysis, hepatocellular injury, and altered iron metabolism. This highlights the multifactorial nature of anemia in CLD and the importance of integrating multiple biomarkers for interpretation.

From a clinical perspective, these findings support a more stratified approach to anemia management in CLD. The combination of biomarkers—such as EPO, STFR, ferritin, WBC, and MCV—together with RBC morphology, may help distinguish underlying mechanisms and guide more targeted therapeutic strategies. For instance, elevated ferritin with high WBC may indicate inflammation-driven anemia, whereas increased STFR with insufficient EPO may suggest impaired erythropoietic compensation (36, 37). Additionally, the correlation between MCV and liver severity scores (MELD, APRI) suggests that MCV may serve as a simple adjunct marker reflecting disease progression. Overall, integrating these biomarkers into clinical decision-making may facilitate a shift from uniform treatment approaches toward more individualized management strategies.

This research has several limitations. As an exploratory, retrospective and single-center study, it is inherently subject to selection bias. The absence of a control group limits the ability to establish causative relationships or define CLD-specific biomarker thresholds. The relatively small sample size, particularly in the microcytic anemia subgroup (*n* = 13), may have reduced statistical power for certain analyses. Moreover, multiple comparisons were performed without adjustment, increasing the risk of type I errors; therefore, the observed correlations should be interpreted as hypothesis-generating. The cohort was predominantly composed of patients with hepatitis B and alcoholic liver disease, underrepresenting other etiologies such as NAFLD and autoimmune hepatitis, which may limit the generalizability of the findings. Finally, although stratification by hepcidin levels could provide additional insights into iron metabolism, the limited number of patients with available hepcidin measurements constrained our ability to perform subgroup analyses with sufficient statistical power. These exploratory findings should be validated in larger, prospective, multi-center studies with well-matched control groups and a more etiologically diverse patient population.

## Conclusion

In conclusion, the exploratory findings propose the framework for a stratified, biomarker-guided approach to anemia in CLD. The specific correlations we identified, such as the EPO-STFR relationship in macrocytic/normocytic anemia and the link between MCV and liver severity scores, provide a pathophysiological rationale for moving beyond uniform treatment. This strategy enhances clinical management by helping to distinguish patients who may benefit from targeted therapies like erythropoiesis-stimulating agents from those where other interventions, such as addressing inflammation, are paramount. These preliminary insights must be confirmed and their clinical applicability assessed in larger, prospective studies.

## Data Availability

The raw data supporting the conclusions of this article will be made available by the authors, without undue reservation.

## Footnotes

1
http://www.R-project.org

## Glossary

AKP: Alkaline phosphatase
ALB: Albumin
ALT: Alanine aminotransferase
ANOVA: Analysis of variance
APRI: Aspartate aminotransferase to platelet ratio index
APTT: Activated partial thromboplastin time
AST: Aspartate aminotransferase
A/G: Albumin globulin ratio
BUN: Blood urea nitrogen
CLD: Chronic liver disease
CR: Creatinine
DBIL: Direct bilirubin
EPO: Erythropoietin
Fe: Iron
FIB: Fibrinogen
FIB-4: Fibrosis-4
FOL: Total folate or folic acid
GGT: Gamma-glutamyl transferase
GLB: Gamma-globulin
HB: Hemoglobin
HCT: Hematocrit test
HP: Haptoglobin
IBIL: Indirect bilirubin
IFAB: Intrinsic factor antibody
IL-1: Interleukin 1
IL-6: Interleukin 6
INR: International normalized ratio
MCH: Mean corpuscular hemoglobin
MCHC: Mean cell hemoglobin concentration
MCV: Mean corpuscular volume
MELD: Model for end-stage liver disease
PCT: Procalcitonin test
PLT: Platelet
PT: Prothrombin time
PTA: Plasma thromboplastin antecedent
RBC: Red blood cell
RDW-CV: Red blood cell distribution width
STFR: Soluble transferrin receptor
TBIL: Total bilirubin
TF: Transferrin
TIBC: Total iron-binding capacity
TNF-*α*: Tumor necrosis factor-alpha
TP: Total protein
UIBC: Unsaturated iron-binding capacity
VitB12: Vitamin B12
WBC: White blood cell count
