# Supplementary figures and images for: Serum biomarker analysis may guide management of anemia in patients with chronic liver disease
Source: Front Med (Lausanne). 2026 Apr 29;13:1797978. doi: 10.3389/fmed.2026.1797978 (PMC13167569; doi:10.3389/fmed.2026.1797978)

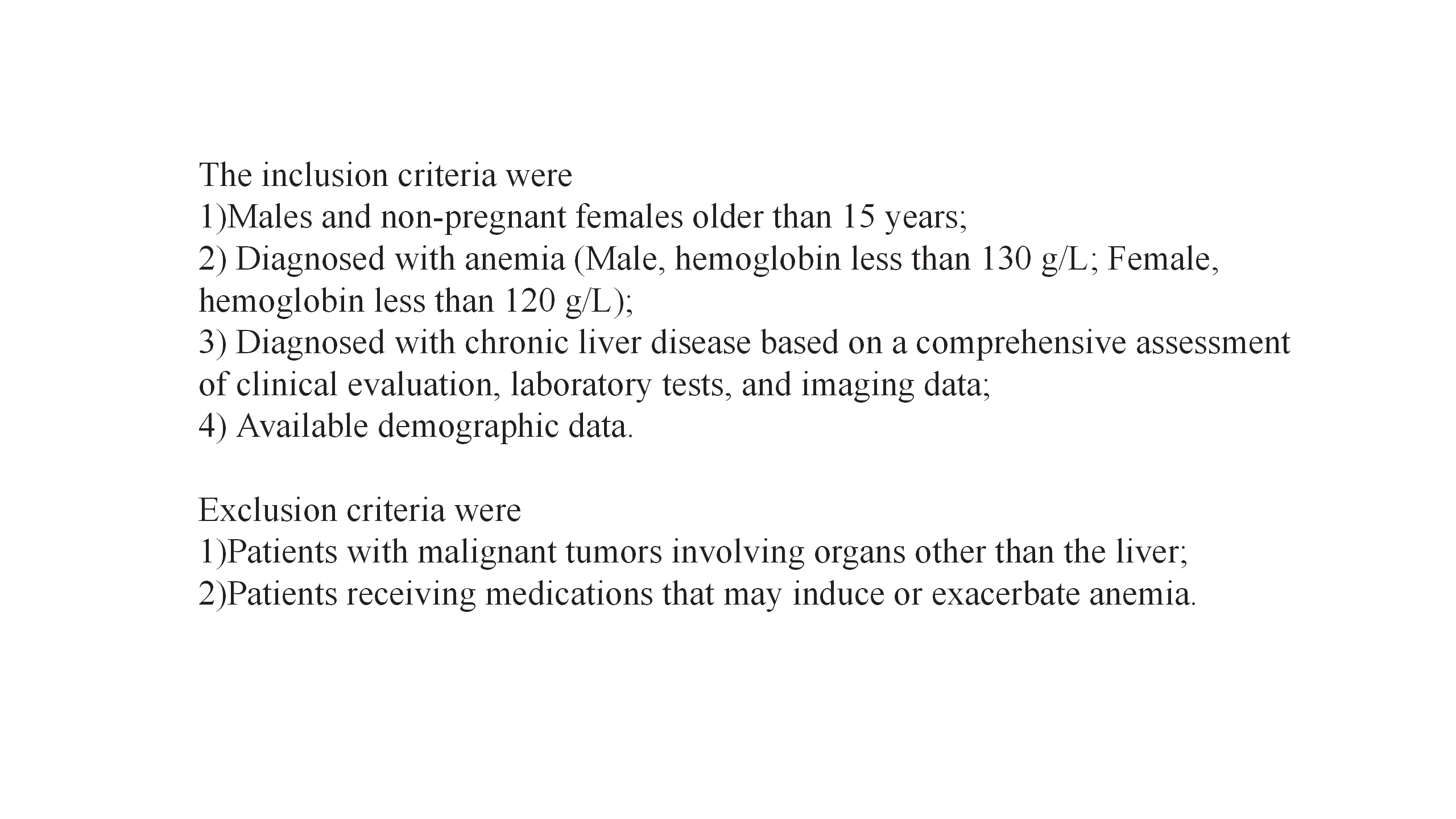

Supplement: Supplementary file 1 [file Image_1.tif]

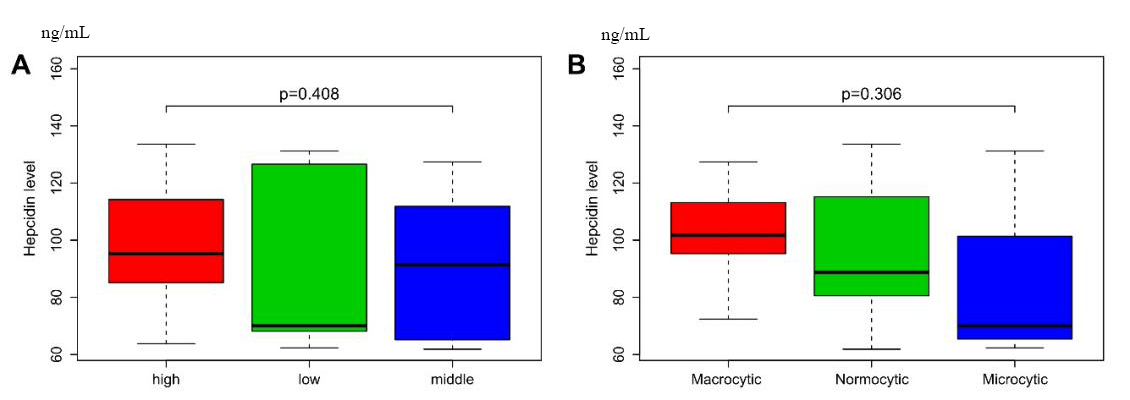

Supplement: Supplementary file 2 [file Image_2.tif]
