# Supplementary material for: Serum biomarker analysis may guide management of anemia in patients with chronic liver disease
Source: Front Med (Lausanne). 2026 Apr 29;13:1797978. doi: 10.3389/fmed.2026.1797978 (PMC13167569; doi:10.3389/fmed.2026.1797978)
Supplement: Supplementary file 3 [file Table_1.docx]

**Supplemental Table 1: Descriptive statistics of the first cohort**

| Descriptive Statistics | | | | |
| --- | --- | --- | --- | --- |
|  | Group | Mean | Std. Deviation | N |
| Bilirubin | 1 | 39.933 | 46.945 | 12 |
|  | 2 | 96.726 | 130.259 | 57 |
|  | 3 | 86.833 | 81.496 | 27 |
|  | Total | 86.845 | 111.406 | 96 |
| INR | 1 | 1.460 | 0.359 | 12 |
|  | 2 | 1.473 | 0.421 | 57 |
|  | 3 | 1.671 | 0.411 | 27 |
|  | Total | 1.527 | 0.417 | 96 |
| CR | 1 | 56.500 | 10.639 | 12 |
|  | 2 | 68.810 | 34.933 | 57 |
|  | 3 | 69.000 | 45.100 | 27 |
|  | Total | 67.320 | 36.139 | 96 |
| PTA | 1 | 64.750 | 13.039 | 12 |
|  | 2 | 63.530 | 20.905 | 57 |
|  | 3 | 52.630 | 17.757 | 27 |
|  | Total | 60.610 | 19.722 | 96 |
| ALB | 1 | 35.500 | 4.009 | 12 |
|  | 2 | 32.391 | 4.981 | 57 |
|  | 3 | 33.485 | 4.070 | 27 |
|  | Total | 33.087 | 4.700 | 96 |
| BUN | 1 | 4.011 | 0.960 | 12 |
|  | 2 | 5.962 | 4.455 | 57 |
|  | 3 | 6.277 | 5.979 | 27 |
|  | Total | 5.807 | 4.699 | 96 |
| PCT | 1 | 0.173 | 0.158 | 12 |
|  | 2 | 0.425 | 0.689 | 57 |
|  | 3 | 0.541 | 1.222 | 27 |
|  | Total | 0.426 | 0.839 | 96 |
| HB | 1 | 79.830 | 14.947 | 12 |
|  | 2 | 99.950 | 16.200 | 57 |
|  | 3 | 97.330 | 16.446 | 27 |
|  | Total | 96.700 | 17.233 | 96 |
| RBC | 1 | 3.671 | 0.648 | 12 |
|  | 2 | 3.213 | 0.518 | 57 |
|  | 3 | 2.730 | 0.516 | 27 |
|  | Total | 3.134 | 0.605 | 96 |
| HCT | 1 | 26.675 | 4.455 | 12 |
|  | 2 | 29.968 | 4.698 | 57 |
|  | 3 | 28.726 | 4.719 | 27 |
|  | Total | 29.207 | 4.757 | 96 |
| MCV | 1 | 72.883 | 5.459 | 12 |
|  | 2 | 93.482 | 5.309 | 57 |
|  | 3 | 105.841 | 4.791 | 27 |
|  | Total | 94.383 | 11.069 | 96 |
| MCH | 1 | 21.783 | 2.304 | 12 |
|  | 2 | 31.207 | 2.591 | 57 |
|  | 3 | 35.830 | 2.099 | 27 |
|  | Total | 31.329 | 4.802 | 96 |
| MCHC | 1 | 298.330 | 13.527 | 12 |
|  | 2 | 333.470 | 14.614 | 57 |
|  | 3 | 338.700 | 15.838 | 27 |
|  | Total | 330.550 | 19.258 | 96 |
| RDW-CV | 1 | 19.667 | 1.925 | 12 |
|  | 2 | 16.481 | 3.481 | 57 |
|  | 3 | 16.633 | 3.666 | 27 |
|  | Total | 16.922 | 3.513 | 96 |
| WBC | 1 | 4.756 | 5.781 | 12 |
|  | 2 | 6.133 | 5.884 | 57 |
|  | 3 | 5.268 | 3.417 | 27 |
|  | Total | 5.718 | 5.268 | 96 |
| PLT | 1 | 100.500 | 99.899 | 12 |
|  | 2 | 124.750 | 90.266 | 57 |
|  | 3 | 76.300 | 45.086 | 27 |
|  | Total | 108.090 | 83.523 | 96 |
| EPO | 1 | 279.211 | 387.413 | 12 |
|  | 2 | 67.855 | 226.174 | 57 |
|  | 3 | 51.614 | 78.577 | 27 |
|  | Total | 89.707 | 233.363 | 96 |
| Fe | 1 | 6.459 | 3.169 | 12 |
|  | 2 | 27.726 | 85.675 | 57 |
|  | 3 | 17.740 | 10.844 | 27 |
|  | Total | 22.259 | 66.449 | 96 |
| UIBC | 1 | 51.708 | 12.176 | 12 |
|  | 2 | 21.388 | 16.365 | 57 |
|  | 3 | 16.037 | 11.524 | 27 |
|  | Total | 23.673 | 18.176 | 96 |
| TIBC | 1 | 58.168 | 13.013 | 12 |
|  | 2 | 38.051 | 12.479 | 57 |
|  | 3 | 33.777 | 10.149 | 27 |
|  | Total | 39.363 | 13.934 | 96 |
| TF | 1 | 2.692 | 0.716 | 12 |
|  | 2 | 3.364 | 12.507 | 57 |
|  | 3 | 1.454 | 0.459 | 27 |
|  | Total | 2.743 | 9.645 | 96 |
| STFR | 1 | 65.713 | 28.534 | 12 |
|  | 2 | 23.625 | 13.970 | 57 |
|  | 3 | 25.747 | 11.567 | 27 |
|  | Total | 29.483 | 20.888 | 96 |
| Ferritin | 1 | 18.982 | 15.240 | 12 |
|  | 2 | 764.813 | 1242.755 | 57 |
|  | 3 | 511.178 | 532.874 | 27 |
|  | Total | 600.249 | 1024.368 | 96 |
| Soluable transferrin index（6.42-22.37） | 1 | 57.729 | 26.840 | 12 |
|  | 2 | 38.042 | 33.070 | 57 |
|  | 3 | 43.923 | 38.654 | 27 |
|  | Total | 42.157 | 34.332 | 96 |
| VitB12（243-894） | 1 | 958.700 | 731.568 | 12 |
|  | 2 | 1519.988 | 1041.149 | 57 |
|  | 3 | 1180.107 | 716.329 | 27 |
|  | Total | 1354.236 | 941.409 | 96 |
| FOL（4.2-19.9） | 1 | 10.023 | 7.575 | 12 |
|  | 2 | 9.060 | 5.232 | 57 |
|  | 3 | 9.466 | 7.327 | 27 |
|  | Total | 9.295 | 6.130 | 96 |
| IFAB | 1 | 1.066 | 0.101 | 12 |
|  | 2 | 1.239 | 1.187 | 57 |
|  | 3 | 1.056 | 0.192 | 27 |
|  | Total | 1.166 | 0.922 | 96 |

**Supplemental Table 2: Correlation between inflammatory factors and hepcidin, ferritin, and anemia types.**

|  | | PCT | WBC | IL-6 | IL-1 | TNF-α |
| --- | --- | --- | --- | --- | --- | --- |
| Ferritin | Correlation | 0.259 | 0.439 | -0.022 | 0.227 | 0.164 |
|  | p-value | 0.134 | 0.008 | 0.898 | 0.190 | 0.347 |
| Hepcidin | Correlation | 0.091 | -0.088 | -0.002 | -0.033 | 0.242 |
|  | p-value | 0.477 | 0.490 | 0.986 | 0.797 | 0.054 |
| Anemia  type | Correlation | 0.003 | 0.044 | 0.081 | 0.159 | 0.106 |
|  | p-value | 0.981 | 0.729 | 0.525 | 0.211 | 0.406 |

**Supplemental Table 3: Descriptive statistics of the second cohort.**

| Descriptive Statistics | | | | |
| --- | --- | --- | --- | --- |
|  | Group | Mean | Std. Deviation | N |
| HB | 1 | 87.000 | 13.653 | 6 |
|  | 2 | 119.430 | 24.080 | 37 |
|  | 3 | 104.500 | 18.971 | 18 |
|  | Total | 111.840 | 24.059 | 61 |
| WBC | 1 | 7.725 | 8.152 | 6 |
|  | 2 | 5.358 | 3.340 | 37 |
|  | 3 | 6.611 | 5.315 | 18 |
|  | Total | 5.961 | 4.571 | 61 |
| RBC | 1 | 3.873 | 0.710 | 6 |
|  | 2 | 4.691 | 5.450 | 37 |
|  | 3 | 2.920 | 0.530 | 18 |
|  | Total | 4.088 | 4.311 | 61 |
| HCT | 1 | 28.883 | 4.037 | 6 |
|  | 2 | 35.278 | 7.078 | 37 |
|  | 3 | 30.972 | 5.060 | 18 |
|  | Total | 33.379 | 6.682 | 61 |
| MCV | 1 | 75.150 | 4.878 | 6 |
|  | 2 | 92.970 | 4.784 | 37 |
|  | 3 | 106.528 | 4.261 | 18 |
|  | Total | 95.218 | 10.130 | 61 |
| MCH | 1 | 22.633 | 1.810 | 6 |
|  | 2 | 31.411 | 2.450 | 37 |
|  | 3 | 35.767 | 1.426 | 18 |
|  | Total | 31.833 | 4.203 | 61 |
| MCHC | 1 | 301.000 | 10.218 | 6 |
|  | 2 | 337.590 | 13.349 | 37 |
|  | 3 | 335.830 | 12.706 | 18 |
|  | Total | 333.480 | 16.702 | 61 |
| PLT | 1 | 104.670 | 70.128 | 6 |
|  | 2 | 111.140 | 48.701 | 37 |
|  | 3 | 86.110 | 48.002 | 18 |
|  | Total | 103.110 | 51.112 | 61 |
| Hepcidin | 1 | 88.660 | 27.030 | 6 |
|  | 2 | 98.700 | 23.970 | 37 |
|  | 3 | 94.650 | 21.700 | 18 |
|  | Total | 96.520 | 23.430 | 61 |
| IL-6 | 1 | 27.526 | 6.224 | 6 |
|  | 2 | 25.314 | 7.029 | 37 |
|  | 3 | 28.065 | 6.292 | 18 |
|  | Total | 26.344 | 6.766 | 61 |
| IL-1 | 1 | 87.317 | 22.263 | 6 |
|  | 2 | 84.315 | 18.774 | 37 |
|  | 3 | 90.261 | 14.670 | 18 |
|  | Total | 86.365 | 17.917 | 61 |
| TNF-α | 1 | 54.215 | 19.697 | 6 |
|  | 2 | 56.984 | 12.342 | 37 |
|  | 3 | 58.219 | 11.870 | 18 |
|  | Total | 57.080 | 12.840 | 61 |
| PT | 1 | 14.367 | 1.308 | 6 |
|  | 2 | 15.557 | 4.333 | 37 |
|  | 3 | 18.583 | 4.047 | 18 |
|  | Total | 16.333 | 4.281 | 61 |
| PT-INR | 1 | 1.302 | 0.130 | 6 |
|  | 2 | 1.401 | 0.388 | 37 |
|  | 3 | 1.658 | 0.338 | 18 |
|  | Total | 1.467 | 0.375 | 61 |
| PTA% | 1 | 67.330 | 10.013 | 6 |
|  | 2 | 66.030 | 18.900 | 37 |
|  | 3 | 51.220 | 13.876 | 18 |
|  | Total | 61.790 | 18.024 | 61 |
| APTT | 1 | 31.867 | 12.322 | 6 |
|  | 2 | 36.305 | 6.226 | 37 |
|  | 3 | 34.381 | 12.664 | 18 |
|  | Total | 35.301 | 9.133 | 61 |
| FIB | 1 | 2.208 | 1.073 | 6 |
|  | 2 | 2.403 | 1.005 | 37 |
|  | 3 | 1.892 | 0.762 | 18 |
|  | Total | 2.233 | 0.959 | 61 |
| ALT | 1 | 30.170 | 20.371 | 6 |
|  | 2 | 67.300 | 103.217 | 37 |
|  | 3 | 30.390 | 13.639 | 18 |
|  | Total | 52.750 | 82.529 | 61 |
| AST | 1 | 48.000 | 23.656 | 6 |
|  | 2 | 103.050 | 129.507 | 37 |
|  | 3 | 59.280 | 29.732 | 18 |
|  | Total | 84.720 | 104.387 | 61 |
| GGT | 1 | 165.670 | 139.759 | 6 |
|  | 2 | 151.160 | 232.817 | 37 |
|  | 3 | 186.720 | 254.417 | 18 |
|  | Total | 163.080 | 229.664 | 61 |
| AKP | 1 | 126.500 | 40.545 | 6 |
|  | 2 | 151.080 | 75.725 | 37 |
|  | 3 | 156.610 | 103.982 | 18 |
|  | Total | 150.300 | 81.915 | 61 |
| TBIL | 1 | 30.900 | 19.920 | 6 |
|  | 2 | 99.084 | 144.534 | 37 |
|  | 3 | 88.694 | 80.609 | 18 |
|  | Total | 89.311 | 121.690 | 61 |
| DBIL | 1 | 12.600 | 5.811 | 6 |
|  | 2 | 69.095 | 111.103 | 37 |
|  | 3 | 56.133 | 57.552 | 18 |
|  | Total | 59.713 | 92.885 | 61 |
| IBIL | 1 | 18.300 | 15.357 | 6 |
|  | 2 | 29.973 | 34.776 | 37 |
|  | 3 | 31.402 | 25.304 | 18 |
|  | Total | 29.246 | 30.666 | 61 |
| TP | 1 | 71.167 | 4.619 | 6 |
|  | 2 | 66.235 | 8.330 | 37 |
|  | 3 | 66.617 | 8.812 | 18 |
|  | Total | 66.833 | 8.217 | 61 |
| ALB | 1 | 36.433 | 2.368 | 6 |
|  | 2 | 34.584 | 6.652 | 37 |
|  | 3 | 32.956 | 3.631 | 18 |
|  | Total | 34.285 | 5.639 | 61 |
| GLB | 1 | 37.433 | 6.931 | 6 |
|  | 2 | 31.592 | 8.780 | 37 |
|  | 3 | 33.644 | 9.144 | 18 |
|  | Total | 32.772 | 8.787 | 61 |
| A/G | 1 | 0.998 | 0.223 | 6 |
|  | 2 | 1.174 | 0.394 | 37 |
|  | 3 | 1.045 | 0.291 | 18 |
|  | Total | 1.119 | 0.356 | 61 |
| BUN | 1 | 4.558 | 0.937 | 6 |
|  | 2 | 5.960 | 4.142 | 37 |
|  | 3 | 4.946 | 4.675 | 18 |
|  | Total | 5.523 | 4.107 | 61 |
| CR | 1 | 62.830 | 11.957 | 6 |
|  | 2 | 72.270 | 43.181 | 37 |
|  | 3 | 66.440 | 48.102 | 18 |
|  | Total | 69.620 | 42.405 | 61 |
| PCT | 1 | 0.124 | 0.079 | 6 |
|  | 2 | 0.963 | 4.632 | 37 |
|  | 3 | 0.283 | 0.252 | 18 |
|  | Total | 0.680 | 3.608 | 61 |

**Supplemental Table 4: ANOVA analysis of the first cohort - Non-significant indicators**

**variances equal**

| Dependent Variable | Type III Sum of Squares | df | Mean Square | F | Sig. |
| --- | --- | --- | --- | --- | --- |
| INR | 0.779 | 2 | 0.390 | 2.302 | 0.106 |
| CR | 1607.112 | 2 | 803.556 | 0.61 | 0.545 |
| ALB | 101.745 | 2 | 50.873 | 2.37 | 0.099 |
| BUN | 46.064 | 2 | 23.032 | 1.044 | 0.356 |
| PCT | 1.124 | 2 | 0.562 | 0.796 | 0.454 |
| HCT | 116.151 | 2 | 58.076 | 2.656 | 0.076 |
| WBC | 26.381 | 2 | 13.190 | 0.470 | 0.626 |
| Fe | 5250.393 | 2 | 2625.197 | 0.589 | 0.557 |
| TF | 66.834 | 2 | 33.417 | 0.354 | 0.703 |
| VitB12 | 4262045.795 | 2 | 2131022.897 | 2.479 | 0.089 |
| FOL | 10.288 | 2 | 5.144 | 0.134 | 0.874 |
| IFAB | 0.752 | 2 | 0.376 | 0.437 | 0.647 |

**Variances unequal**

| Dependent Variable | Statistic | df1 | df2 | Sig. |
| --- | --- | --- | --- | --- |
| EPO | 2.006 | 2 | 29.022 | 0.153 |
| Ferritin | 0.685 | 2 | 32.171 | 0.511 |

**Supplemental Table 5: Expression of various indicators when grouped by MCV - Non-significant indicators**

**Equal variances**

| Dependent Variable | Type III Sum of Squares | df | Mean Square | F | Sig. |
| --- | --- | --- | --- | --- | --- |
| RBC | 38.29 | 2 | 19.145 | 1.031 | 0.363 |
| PLT | 7598.761 | 2 | 3799.381 | 1.478 | 0.237 |
| Hepcidin | 609.967 | 2 | 304.984 | 0.547 | 0.582 |
| IL-6 | 100.897 | 2 | 50.449 | 1.106 | 0.338 |
| IL-1 | 434.152 | 2 | 217.076 | 0.669 | 0.516 |
| APTT | 123.345 | 2 | 61.672 | 0.733 | 0.485 |
| FIB | 3.163 | 2 | 1.581 | 1.764 | 0.180 |
| ALT | 19890.471 | 2 | 9945.235 | 1.484 | 0.235 |
| GGT | 15356.619 | 2 | 7678.309 | 0.141 | 0.868 |
| AKP | 4138.154 | 2 | 2069.077 | 0.301 | 0.741 |
| IBIL | 822.088 | 2 | 411.044 | 0.429 | 0.653 |
| TP | 126.752 | 2 | 63.376 | 0.937 | 0.398 |
| GLB | 195.597 | 2 | 97.799 | 1.278 | 0.286 |
| A/G | 0.298 | 2 | 0.149 | 1.186 | 0.313 |
| BUN | 18.643 | 2 | 9.321 | 0.544 | 0.583 |
| CR | 717.753 | 2 | 358.876 | 0.194 | 0.824 |
| PCT | 7.659 | 2 | 3.830 | 0.287 | 0.751 |

**Unequal Variances**

| Dependent Variable | Statistic | df1 | df2 | Sig. |
| --- | --- | --- | --- | --- |
| WBC | 0.545 | 2 | 11.692 | 0.594 |
| TNF-α | 0.322 | 2 | 12.654 | 0.731 |
| AST | 3.169 | 2 | 28.734 | 0.057 |
| ALB | 3.149 | 2 | 20.736 | 0.064 |

**Supplemental Table 6: Expression of various indicators when grouped by bilirubin level - Non-significant indicators**

**Equal variances**

| Dependent Variable | Type III Sum of Squares | df | Mean Square | F | Sig. |
| --- | --- | --- | --- | --- | --- |
| WBC | 28.574 | 2 | 14.287 | 0.673 | 0.514 |
| RBC | 5.649 | 2 | 2.824 | 0.145 | 0.865 |
| MCV | 464.304 | 2 | 232.152 | 2.433 | 0.097 |
| PLT | 1291.022 | 2 | 645.511 | 0.241 | 0.787 |
| IL-6 | 44.647 | 2 | 22.324 | 0.480 | 0.621 |
| IL-1 | 1438.085 | 2 | 719.043 | 2.301 | 0.109 |
| TNF-α | 166.037 | 2 | 83.019 | 0.502 | 0.608 |
| APTT | 57.604 | 2 | 28.802 | 0.333 | 0.718 |
| GGT | 97767.802 | 2 | 48883.901 | 0.909 | 0.409 |
| TP | 101.105 | 2 | 50.552 | 0.730 | 0.486 |
| ALB | 73.254 | 2 | 36.627 | 1.144 | 0.326 |
| A/G | 0.423 | 2 | 0.211 | 1.696 | 0.193 |
| PCT | 20.27 | 2 | 10.135 | 0.760 | 0.473 |

**Unequal variances**

| Dependent Variable | Statistic | df1 | df2 | Sig. |
| --- | --- | --- | --- | --- |
| HB | 1.062 | 2 | 31.062 | 0.358 |
| HCT | 1.905 | 2 | 32.368 | 0.165 |
| Hepcidin | 0.102 | 2 | 31.694 | 0.903 |
| GLB(g/L) | 3.135 | 2 | 36.872 | 0.055 |
| BUN(mmol/L) | 1.114 | 2 | 40.178 | 0.338 |
| CR | 1.308 | 2 | 38.843 | 0.282 |

**Supplemental Table 7:** **Expression of various indicators when grouped by cytopenia patterns - Non-significant indicators**

**Equal Variance**

| Dependent Variable | Type III Sum of Squares | df | Mean Square | F | Sig. |
| --- | --- | --- | --- | --- | --- |
| Fe | 113.665 | 2 | 56.833 | 0.560 | 0.577 |
| STFR | 746.298 | 2 | 373.149 | 1.752 | 0.190 |
| Ferritin | 5125792.162 | 2 | 2562896.081 | 1.762 | 0.188 |
| VitB12 | 4455708.969 | 2 | 2227854.484 | 1.984 | 0.154 |
| FOL | 85.935 | 2 | 42.968 | 0.534 | 0.592 |
| HB | 141.626 | 2 | 70.813 | 0.375 | 0.690 |
| Hepcidin | 1081.217 | 2 | 540.608 | 1.037 | 0.366 |
| IL-1 | 31.622 | 2 | 15.811 | 0.053 | 0.948 |
| TNF-α | 29.169 | 2 | 14.585 | 0.081 | 0.922 |
| PT | 81.556 | 2 | 40.778 | 2.246 | 0.122 |
| PT-INR | 0.556 | 2 | 0.278 | 1.902 | 0.166 |
| PTA | 845.655 | 2 | 422.827 | 1.391 | 0.263 |
| APTT | 41.955 | 2 | 20.977 | 0.231 | 0.795 |
| ALT | 11438.498 | 2 | 5719.249 | 0.557 | 0.578 |
| AST | 11948.126 | 2 | 5974.063 | 0.382 | 0.686 |
| GGT | 23218.939 | 2 | 11609.469 | 0.455 | 0.639 |
| AKP | 7841.07 | 2 | 3920.535 | 0.512 | 0.604 |
| TP | 39.449 | 2 | 19.725 | 0.281 | 0.757 |
| ALB | 52.289 | 2 | 26.145 | 1.858 | 0.172 |
| GLB | 103.506 | 2 | 51.753 | 0.648 | 0.530 |
| A/G | 0.169 | 2 | 0.084 | 0.780 | 0.467 |
| BUN | 45.194 | 2 | 22.597 | 1.360 | 0.271 |
| CR | 5648.948 | 2 | 2824.474 | 1.023 | 0.371 |

**Unequal Variance**

| Dependent Variable | Statistic | df1 | df2 | Sig. |
| --- | --- | --- | --- | --- |
| EPO | 2.088 | 2 | 19.877 | 0.150 |
| TBIL | 3.793 | 2 | 12.845 | 0.051 |
| DBIL | 3.826 | 2 | 12.693 | 0.050 |
| IBIL | 3.019 | 2 | 14.638 | 0.080 |
